# Data on the determinants of the risk of fatalities, serious injuries and light injuries in traffic accidents on interurban roads in Spain

**DOI:** 10.1016/j.dib.2018.04.117

**Published:** 2018-05-01

**Authors:** María Pilar Sánchez González, Francisco Escribano Sotos, Ángel Tejada Ponce

**Affiliations:** Faculty of Economic and Business Sciences (University of Castilla-La Mancha), Spain

**Keywords:** Risk of fatality, Risk of injury, Panel data, Spain

## Abstract

This article describes the data collection used to analyse the risk of fatalities and injuries resulting from traffic accidents on interurban roads in the provinces of Spain from 1999 to 2015. The database includes data on different factors related to accidents rates for each Spanish province. These data were used in the article entitled “Impact of provincial characteristics on the number of traffic accident victims on interurban roads in Spain” (Sánchez et al., 2018) [1].

## Specifications Table

**Table:** 

Subject area	*Social Sciences*
More specific subject area	*Road Traffic Accidents*
Type of data	*Excel files*
How data was acquired	*Data compilation from public sources*
Data format	*Raw and analysed*
Experimental factors	*Compilation of information on the number of victims according to the seriousness of the injury and other factors related to road accident statistics for each Spanish province between 1999 and 2015. Construction of variables and configuration of the database using Excel. Specification and development of econometric models using STATA software.*
Experimental features	*The risks of fatalities, serious injuries and slight injuries and their determining factors for each Spanish province were obtained by means of econometric models with data panel.*
Data source location	*Spanish provinces*
Data accessibility	*Mendeley Data. Number identification (DOI):* 〈http://dx.doi.org/10.17632/yw84spgpmc.3〉
	〈https://data.mendeley.com/datasets/yw84spgpmc/3〉 [2]

## Value of the data

• The database developed provides the scientific community with a complete and diverse set of determinants of traffic accidents on interurban roads in Spain's smallest territorial units, the provinces.
• The compilation of the data on the provincial variables selected has important implications for the future of traffic accident research in Spain, given the scant literature available using provinces as the unit of analysis.
• The data have the potential to serve as guidance for future research in which the objective is a more specific analysis of traffic accident rates in other territorial units or countries.
• The database pools data gathered from different database, thus providing a useful tool for the research community with an interest in traffic accidents.

## 1 Data

The data presented in this summary were used to develop the research conducted by Sánchez et al. [1]. The data were taken from statistical information compiled in Spain by Directorate General for Traffic, the National Statistics Institute, the Ministry of the Interior, the Ministry of Public Works and Transport and the Ministry of Agriculture, Fisheries, Food and Environment. All the observations of the dependent variables (risks of fatality and injury) were gathered for each province for the 1999–2015 period. The 850 observations and the database design enabled us to conduct an econometric panel data analysis. The record of the number of victims of traffic accidents classified according to seriousness of injury by the Directorate General for Traffic facilitated our twofold objective:

• To quantify the risk of fatality, serious injury and slight injury for each Spanish province resulting from the specific characteristics of each province.
• To determine the relationship between each explanatory variable and the risks of fatality, serious injury and slight injury.

## 2 Experimental design, materials and methods

Once we had the data for the specific set of individual units (provinces) and observations for these units over a defined period of time (1999–2015), we constructed the database by means of panel data. To this end, the columns express each of the variables selected for the main research or those necessary to derive them. The rows include all the observations for each province and in each year of the study period. The provinces are organized in alphabetical order according to the Autonomous Community of which they form part.

The first four columns of the Excel workbook were used to encode the observations: province (name of province); time (year of observation); cprovince (value between 1 and 50 to encode each province) and ctime (value between 1 and 17 to encode the year of observation).

The risks of fatality, serious injury and slight injury for each province in the period under study (dependent variables) were obtained by means of the relationship between two variables: the number of fatalities, serious injuries and slight injuries on interurban roads [3] and the millions of vehicle-kilometres travelled on the same roads [4]. This definition of the dependent variables enabled us to mitigate the heterogeneity across the provinces with respect to the number of vehicle-kilometres travelled on their roads.

The database also includes the different independent variables used in the main research or those necessary to derive them. The compilation and/or the construction of some of the determinants required taking into account certain considerations.

The penalty-points driving licence came into force on 1 July 2006 [5]. The annual nature of the observations for each province gave rise to the question of what value to attribute to the dummy variable included to examine the effect of the introduction of the penalty-points driving licence system. Following a review of the related literature, we assigned a value of 1 to this variable in 20,006 in all the provinces, based on the results of previous studies suggesting that the measure had an impact before its implementation.

To construct the variables referring to the sociodemographic aspects of the models (annual variation in population density and motorization rate), we used the population of each province [6]. This information is published by the National Statistics Institute as of 1 January each year. As some of the variables referred to 31 December, for each year we decided to use the population as of 1 January of the following year, so as to achieve greater temporal homogeneity across the variables.

Some of the factors included in the database are missing certain observations. First, the climate records published in the Annual Statistics of the Ministry of Agriculture, Fisheries, Food and Environment [7] did not provide data for all the provinces across all the years of the study period. Hence, this variable includes only 828 observations of the total of 850.

The non-availability of data is also present in the unemployment rate variable [8]. However, in this case, it is due to a lack of data for 199 and 2000 as a result of the new of the new definition of unemployed established in Commission Regulation (EC) no. 1897/2000. As suggested by the National Statistics Institute, the figures for 1999 and 2000 are not directly comparable to the other years of the study period.

The calculation of the absolute annual variation in population density (difference between the population density of a province in a particular year and that of the preceding year) resulted in a lack of information for 1999 for all the provinces since no data were available for this variable referring to 1998.

The non-availability in the three series of all the observations had no substantial impact mainly because the models include the investment in infrastructure, lagged one and two periods. STATA did not utilize the data for 1999 and 2000 as there were no lagged observations for these years.

Investments in infrastructure (replacement and construction) per kilometre of road were constructed from two variables. The first is the investment in replacement or construction of interurban roads in each province, regardless of the authority responsible for maintaining the network [4]. The observations of this variable are expressed at constant 2015 prices using the data series from consumer price index of each province [9]. The second of the variables used to construct the two infrastructure variables is the total number of kilometres in the interurban road network of each province [4].

The traffic volume variable, which was included to measure the effect of exposure on the risk of fatalities and injuries in traffic accidents, was obtained using the information published by the Ministry of Public Works and Transport with respect to the number of kilometres travelled on interurban roads (millions of vehicle-kilometres) and the number of kilometres of interurban roads in each province [4]. The Ministry calculates the indicator “millions of vehicle-kilometres travelled (*veh-km*)” using the following mathematical expression:

$$veh-km=\frac{365\cdot ADT\cdot L_{i}}{10^{6}}$$

where *ADT* is the mean daily intensity of traffic and *L_i_* the length of the stretch of interurban road.

Using this expression and the information available in our database for the variables “*veh-km*” y “*L_i_*”, we obtained the traffic volume variable (*ADT*).
