# Supplementary material for: Data on the determinants of the risk of fatalities, serious injuries and light injuries in traffic accidents on interurban roads in Spain
Source: Data Brief. 2018 May 1;18:1941–4. doi: 10.1016/j.dib.2018.04.117 (PMC5998648; doi:10.1016/j.dib.2018.04.117)
Supplement: Supplementary file 1 — Supplementary material [file mmc1.docx]

**Conflict of Interest**

The authors whose names are listed below certifate that they have no conflict of interest. María Pilar Sánchez González; Angel Tejada Ponce and Francisco Escribano Sotos.
